# Resveratrol and N-acetylcystein reduce hepatic steatosis but enhance initiation and progression of hepatocellular carcinoma by inhibiting GST-pi-MAPK axis in mice

**DOI:** 10.3389/fphar.2025.1574039

**Published:** 2025-04-28

**Authors:** Mi Zhang, Weigang Yuan, Chun Li, Chanyuan Chen, Xiang Liu, Zhilu Ma, Yifei Xiang, Guisha Chen, Chunxu Wang, Lei Li, Lingli Wang, Zhong Xu, Chuanrui Xu

**Affiliations:** ^1^ School of Pharmacy, Tongji Medical College, Huazhong University of Science and Technology, Wuhan, China; ^2^ School of Pharmacy, Lanzhou University, Lanzhou, China; ^3^ Department of Clinical Laboratory, Xianning Central Hospital, The First Affiliated Hospital of Hubei University of Science and Technology, Xianning, China; ^4^ Department of Pharmacy, Union Hospital, Tongji Medical College, Huazhong University of Science and Technology, Wuhan, China; ^5^ School of Pharmaceutical Sciences, Guangzhou University of Chinese Medicine, Guangzhou, China; ^6^ School of Basic Medicine, Tongji Medical College, Huazhong University of Science and Technology, Wuhan, China; ^7^ Department of Gastroenterology and Hubei Clinical Center and Key Lab of Intestinal and Colorectal Diseases, Zhongnan Hospital of Wuhan University, Wuhan, China; ^8^ Health Management Center, Zhongnan Hospital of Wuhan University, Wuhan, China; ^9^ Department of Pharmacy, Tongren Polytechnic College, Tongren, China

**Keywords:** antioxidant, hepatocellular carcinoma, resveratrol, N-acetylcystein, GST-Pi

## Abstract

**Introduction:**

Accumulating evidence indicates that antioxidants promote tumor growth and metastasis after tumor onset in several cancer types. However, whether antioxidants prevent or accelerate hepatic tumorigenesis during steatosis remains unknown. Therefore, we investigated the effects of resveratrol (RES) and N-acetylcysteine (NAC) on hepatocellular carcinoma (HCC) development using two fatty liver mouse models.

**Methods:**

High-fat diet (HFD) plus diethylnitrosamine (DEN)- and AKT/Ras-induced primary HCC mouse models were used. The weight, liver weight ratio and the number of HCC tumors were calculated and histological features of mouse HCC tissues were analyzed using immumohistochemical staining such as hematoxylin and eosin staining. Proteomic analysis was used to screen for differences in liver cancer progression between antioxidant-treated HCC and models. Protein inhibitor recovery experiments in mice and *in vitro* cells validate the targets screened by proteomic analysis. The expression of GST-pi, p-JNK and p-p38 signaling molecules in HCC were investigated using Western blotting.

**Results:**

RES and NAC enhance HCC formation in both DEN/HFD and AKT/Ras mice. RES and NAC alleviate hepatosteatosis, and reduce ROS and DNA damage in mice. Proteomic analysis and protein inhibitor recovery assay demonstrated that GST-pi is a therapeutic target for antioxidant-induced hepatocellular carcinoma growth. Mechanistically, RES and NAC decreased p-JNK and p-p38, the two major mitogen-activated protein kinases, in HCC cells. Blockade of GST-pi abrogated the reduction in p-JNK and p-p38 levels and increased apoptosis of HCC cells.

**Conclusion:**

Antioxidants may increase the incidence of HCC in a population with fatty liver, despite reduction in ROS production, by inhibiting GST-pi-MAPK axis.

## 1 Introduction

Hepatocellular carcinoma (HCC) accounts for 75%–85% of primary liver cancers and is the third leading cause of cancer-related mortality worldwide (Sung et al., 2021). The primary risk factors associated with HCC are chronic hepatitis B/C virus (HBV/HCV) infection and non-alcoholic fatty liver disease (NAFLD) (Calle and Kaaks, 2004; Taubes, 2012). In recent years, NAFLD has become the most important pathogenic factor of HCC owing to increasing incidence of NAFLD and decreasing incidence of new HBV/HCV infections (Vernon et al., 2011; Huang et al., 2021). The cellular and molecular mechanisms underlying NAFLD-induced HCC are complex and include inflammation, immune response, DNA damage, and oxidative stress (Anstee et al., 2019). NAFLD-related steatosis produces a large number of reactive oxygen species (ROS) that cause hepatocyte damage, liver tissue inflammation, and occurrence of apoptosis and liver regeneration, thus contributing to HCC development (Kawada and Otogawa, 2007; Cohen et al., 2011; Podrini et al., 2013).

Antioxidants have long been used as natural dietary agents to prevent aging and cancer because of their ability to eliminate ROS or other free radicals that cause DNA damage (Morselli et al., 2010; Chandrashekara and Shakarad, 2011; Liu et al., 2018; Russo et al., 2017; Willcox et al., 2004). In the late 1980s to mid-1990s, several studies suggested that consuming a diet rich in vitamin E, vitamin C, or β-carotene could lead to increased plasma concentrations of these vitamins and prevent cancers (Eichholzer et al., 1992; Zhang et al., 1999; Tamimi et al., 2005; Sablina et al., 2005; Godic et al., 2014; Westerlund et al., 2011). Despite the initial indications of potential benefits, large-scale randomized clinical trials have yielded unexpected negative outcomes, and some studies have even suggested that antioxidants could increase the risk of cancer development (Alpha-Tocopherol Beta Carotene Cancer Prevention Study Group, 1994; van Zandwijk et al., 2000; Klein et al., 2011). Studies conducted in mice have indicated that vitamin E and N-acetylcysteine (NAC) accelerate human lung cancer cell growth by reducing ROS, DNA damage, and p53 (Sayin et al., 2014). In mice, the vitamin E analog Trolox and NAC enhanced the invasion and migration of human melanoma cells (Le Gal et al., 2015). These studies indicate that tumor cells may benefit from low ROS levels induced by dietary antioxidants.

Wang et al. found that non-mitochondria-targeting antioxidants, such as NAC and Trolox, prevented hepatic tumorigenesis, whereas mitochondria-targeting antioxidants, such as SS-31 and Mito-Q, accelerated HCC (Wang B. B. et al., 2018). However, they examined the effects of these antioxidants in chemical carcinogen-induced HCC mouse models without steatosis. Whether the preventive use of antioxidants would accelerate or delay HCC formation in a fatty liver context is still unknown. Hepatic steatosis is a known risk factor for HCC, and some antioxidants have been reported to inhibit lipid accumulation in the liver. Therefore, we investigated whether these antioxidants prevented or delayed HCC formation by reducing lipid accumulation in the liver. Antioxidants, including NAC and resveratrol (RES), reduce lipid accumulation and peroxidation in the liver (Ma et al., 2016; Llovet et al., 2015). Given that fatty liver is associated with the development of HCC, we speculate whether antioxidants would prevent the development of NAFLD-related HCC. Therefore, this study aimed to investigate the effects of NAC and RES on HCC initiation and progression in an obesity-related setting.

## 2 Materials and methods

### 2.1 Materials

NAC (purity ≥99%, CAS No. 616-91-1) was obtained from Sigma-Aldrich (St. Louis, MO, United States), and RES (purity ≥99%, CAS No. 501-36-0) was obtained from Zhejiang Great Forest Biomedical Ltd. (Hangzhou, Zhejiang, China). High-fat diet (HFD) chow (CAS No. H10060), normal chow (CAS No. H10010), and RES mixed with normal chow and HFD chow were procured from Beijing Huafukang Bioscience Technology (Beijing, China). The feed formulas used in the experiments are listed in Supplementary Table S1. The glutathione-S-transferase-pi (GST-pi) inhibitor ethacrynic acid (EA) was purchased from BioVision (Milpitas, CA, United States). Sodium carboxymethylcellulose (CMC-Na) was obtained from Sinopharm (Shanghai, China). Unless otherwise specified, all other reagents were purchased from Sigma-Aldrich.

### 2.2 Mice

The Hubei Provincial Center for Disease Control and Prevention provided C57BL/6J mice, and the Beijing Huafukang Bioscience Technology supplied the FVB mice (age: 6-week, weight: 16 g). The protocols for the maintenance, feeding, and handling of all mice were approved by the Animal Experiments Ethical Committee of Huazhong University of Science and Technology. Mice were sacrificed using CO_2_ asphyxiation and subsequent cervical dislocation to reduce animal suffering according to ethical guidelines approved by the Animal Experiments Ethical Committee of Huazhong University of Science and Technology.

### 2.3 DEN/HFD-induced HCC model and treatment

To establish a diethylnitrosamine (DEN)/HFD-induced HCC model, male C57BL/6J mice aged 18 days were first administered DEN (25 mg/kg) via i.p. injection and then repeatedly injected with a second dose of DEN 1 week later (25 mg/kg) (Park et al., 2010). The mice were switched to HFD 3 weeks after the injection and randomized into four groups with 11 mice in each group: normal, DEN/HFD + vehicle, DEN/HFD + RES, and DEN/HFD + NAC. Vehicle-treated HCC model mice were fed HFD chow and drinking water. The DEN/HFD + RES group received RES mixed with HFD chow at 0.4% w/w (Soyoung et al., 2011). The DEN/HFD + NAC group received NAC dissolved in drinking water at 1 g/L (Sayin et al., 2014). The mice were fed HFD chow for 24 weeks and weighed every 2 weeks. Finally, the mice were euthanized, and their livers were weighed, photographed, and collected for subsequent analysis. The number and size of tumor nodules were determined; tumor volume was calculated using the following formula: $V=\text{length}\times {\text{width}}^{2}\times 0.5$ .

### 2.4 AKT/Ras-induced HCC mouse model and treatment

AKT/Ras mouse model was established through tail vein hydrodynamic injection as previously described (Lee et al., 2008; Ho et al., 2012; Chen and Calvisi, 2014). One week after plasmid (pT3-EF1α-myr-AKT: pCaggs-NRasV12: pCMV-SB = 5:5:1 μg per mouse) injection, the mice were fed normal chow and randomized into four groups with four mice in each group: normal, AKT/Ras + vehicle, AKT/Ras + RES, and AKT/Ras + NAC. Vehicle-treated mice were fed normal chow and drinking water. In another AKT/Ras induced HCC mouse model experiment, the AKT/Ras mice were fed normal chow and randomized into five groups with seven mice in each group: AKT/Ras + vehicle, AKT/Ras + RES, AKT/Ras + RES + EA, AKT/Ras + NAC and AKT/Ras + NAC + EA. The AKT/Ras + RES group received RES mixed with normal chow at 0.4% w/w, and the AKT/Ras + NAC group received NAC dissolved in drinking water at 1 g/L. The mice were treated with the GST-pi inhibitor EA (25 mg/kg/day) (Zhang et al., 2021; Madala et al., 2017) in 0.5% CMC-Na by gavage daily until the end of the study. The mice were weighed every 4 days and sacrificed 5 weeks after plasmid injection.

### 2.5 Histological and immunohistochemical analyses

The experimental mice were euthanized in a humane manner, and their livers were dissected and extracted. The livers were washed twice with phosphate-buffered saline by immersion. Liver samples were preserved for protein extraction by snap-freezing on dry ice. The samples were fixed overnight in 4% paraformaldehyde or Tissue-Tek OCT compound (Sakura Finetek, Tokyo, Japan) to prepare paraffin or frozen blocks, respectively. The paraffin-embedded tissues were cut into 5-μm sections for hematoxylin and eosin (H&E), Ki67 (1:100; Cell Signaling, Danvers, MA, United States), and γH2AX (1:800; Abcam, Cambridge, United Kingdom) staining, following previously described methods (Sjogren et al., 2007). Frozen tissue sections were stained with Oil Red O (ORO; Biosharp, Hefei, Anhui, China) for lipid detection and dihydroethidine (DHE; Sigma-Aldrich) for ROS detection, following the manufacturer’s instructions, and the images were captured and analyzed using inverted microscopy (CKX53; Olympus, Shinjuku-ku, Tokyo, Japan).

### 2.6 Serum biochemical and lipid peroxidation assays

The blood samples were collected from the heart and kept at 4°C for 2 h. Next, the blood was centrifuged at 3,000 revolutions per minute (rpm) for 10 min and stored at −80°C. The concentrations of serum triglyceride (TG), total cholesterol (TC), alanine aminotransferase (ALT), and aspartate aminotransferase (AST) were assessed using an automatic biochemical analyzer (Cobas-8,000; Roche, Basel, Switzerland). The lipid peroxidation indexes of malondialdehyde (MDA, A003-4), ROS (E004), lipid peroxidation (LPO, A106-1), total antioxidant capacity (T-AOC, A015-1), and GSH/GSSG (A016-1) were assayed using the respective kits (Nanjing Jiancheng Bioengineering Institute, Nanjing, Jiangsu, China) in accordance with the manufacturer’s instructions.

### 2.7 Cell culture, cell viability, EdU assay and TUNEL assay

The human HCC cell line HepG2 was purchased from the China Center for Type Culture Collection (Shanghai, China). The HepG2 cell line was authenticated by single-tandem repeat profiling and tested for *mycoplasma* contamination. The cells were cultured in Dulbecco’s modified Eagle medium supplemented with 10% fetal bovine serum (Gibco), 100 μg/mL streptomycin, and 100 U/mL penicillin in a humidified incubator at 37°C in the presence of 5% CO_2_. For cell viability analysis, a total of 5,000 cells were seeded into 96-well plates in quadruplicate and cultured for 24 h. Then, the cells were treated with oleic acid (OA; 0.8 mM) for 24 h with or without 25 μM RES (Park et al., 2012; Zhang et al., 2015) or 250 μM NAC (Sayin et al., 2014) and/or GST-pi inhibitor 20 μM EA. After the treatment, cell viability was determined using cell counting kit-8 (CCK-8; Dojindo, Kumamoto Prefecture, Japan). 5-ethynyl-2′-deoxyuridine (EdU) assay was performed using BeyoClick™ EdU cell proliferation kits with Alexa Fluor 488 labeling (Beyotime, Shanghai, China). Terminal deoxynucleotidyl transferase dUTP nick end labeling (TUNEL) was performed using Click-iT™ TUNEL colorimetric detection kit (Thermo Fisher Scientific, Waltham, MA, United States). All experiments were repeated three times.

### 2.8 Western blot analysis

HCC tissue nuclear protein extraction was performed using a nuclear protein extraction kit (Beyotime). RIPA buffer (R0278; Sigma-Aldrich) was used to extract proteins from the liver or HCC tissue via whole cell lysis. The resulting lysates were centrifuged at 12,000 rpm for 10 min, and the supernatants were collected quantitatively. BCA protein assay kit (Beyotime) was used to measure the protein concentration in the lysates. Subsequently, proteins of different molecular sizes were separated on a 10% polyacrylamide gel and electrotransferred onto a polyvinylidene difluoride (PVDF) membrane. After blocking for 1.5 h using 5% skim milk or TBST (TBS with 0.1% Tween-20) containing 5% bovine serum albumin, the membrane was incubated with monoclonal antibodies, washed thrice with TBST, and incubated with the secondary antibodies for 1 h at room temperature. Finally, the membrane was rinsed and developed using an enhanced chemiluminescence system following the manufacturer’s instructions (Perkin Elmer, Waltham, MA, United States). The antibodies used in the experiment are listed in Supplementary Table S2.

### 2.9 Proteomic analysis

Liver or tumor tissues were mixed with SDT buffer containing 100 Mm Tris (hydroxymethyl)aminomethane hydrochloride (Tris-HCl), 4% Sodium dodecyl sulfate (SDS), and 1 mM dithiothreitol (DTT) at pH 7.6. The mixture was then transferred to 2-mL tubes with quartz sand and homogenized twice using an MP homogenizer for 60 s at a speed of 6.0 m/s. After homogenization, the mixture was sonicated and boiled at 100°C for 15 min. The resulting mixture was then centrifuged at 14,000 × *g* for 40 min, and the supernatant was removed and filtered through a 0.22-µm filter. After filtration, the protein content in the supernatant was quantified using BCA protein assay kit, and the samples were stored at −80°C until further use. For additional analysis, 20 µg of protein was mixed with loading buffer and heated at 95°C in a water bath for 5 min. To separate the proteins, 12.5% sodium dodecyl sulfate-polyacrylamide gel electrophoresis was performed at a constant current of 14 mA for 90 min. Protein bands were visualized by staining with Coomassie Blue R-250. Finally, all samples were subjected to filter aided sample preparation process following an established protocol (Wisniewski et al., 2009), and the resulting peptides were collected as filtrates. Subsequently, the peptide mixture from each sample (100 μg) was labeled with tandem mass tag (TMT) reagent according to the instructions provided by the manufacturer (Thermo Fisher Scientific). The digested samples labeled with TMT were fractionated into 10 parts using the Pierce High pH Reversed-Phase Fractionation Kit (Thermo Fisher Scientific), using a method that involves increasing acetonitrile step-gradient elution. The obtained peptides were introduced into a trap column of reverse-phase chromatography (Thermo Scientific Acclaim PepMap100, 100 μm × 2 cm, nanoViper C18) that was linked to the analytical column of C18-reversed phase chromatography (Thermo Scientific Easy Column, 10 cm in length, 3 μm resin) using buffer A consisting of 0.1% formic acid. The peptides were subjected to gradient elution with buffer B consisting of 84% acetonitrile and 0.1% formic acid. Gradient elution was performed in three steps: 0%–55% over 80 min, 55%–100% over 5 min, and 100% for 5 min. Using Easy-nLC system (Thermo Fisher Scientific), eluted peptides were introduced into Q Exactive mass spectrometer (Thermo Fisher Scientific) for 90 min. The mass spectrometer was operated in data-dependent mode, with one MS scan followed by 20 MS/MS scans per cycle. Peptides were searched using the MASCOT engine (version 2.2) integrated into Proteome Discoverer (version 1.4; Thermo Fisher Scientific) to query the database. For bioinformatics analysis, gene ontology (GO) annotation, Kyoto Encyclopedia of Genes and Genomes (KEGG) pathway annotation, functional enrichment analysis, and hierarchical clustering were performed.

### 2.10 Statistical analysis

All statistical analyses were performed using GraphPad Prism 7.0. Data were expressed as the mean ± SD, and the groups were compared for significant differences using ANOVA followed by Dunnett’s *t*-test. *P* < 0.01 and *P* < 0.05 were considered very significant and significant, respectively.

## 3 Results

### 3.1 RES and NAC accelerate HCC initiation and development in both DEN/HFD and AKT/Ras mice

**FIGURE 1:**
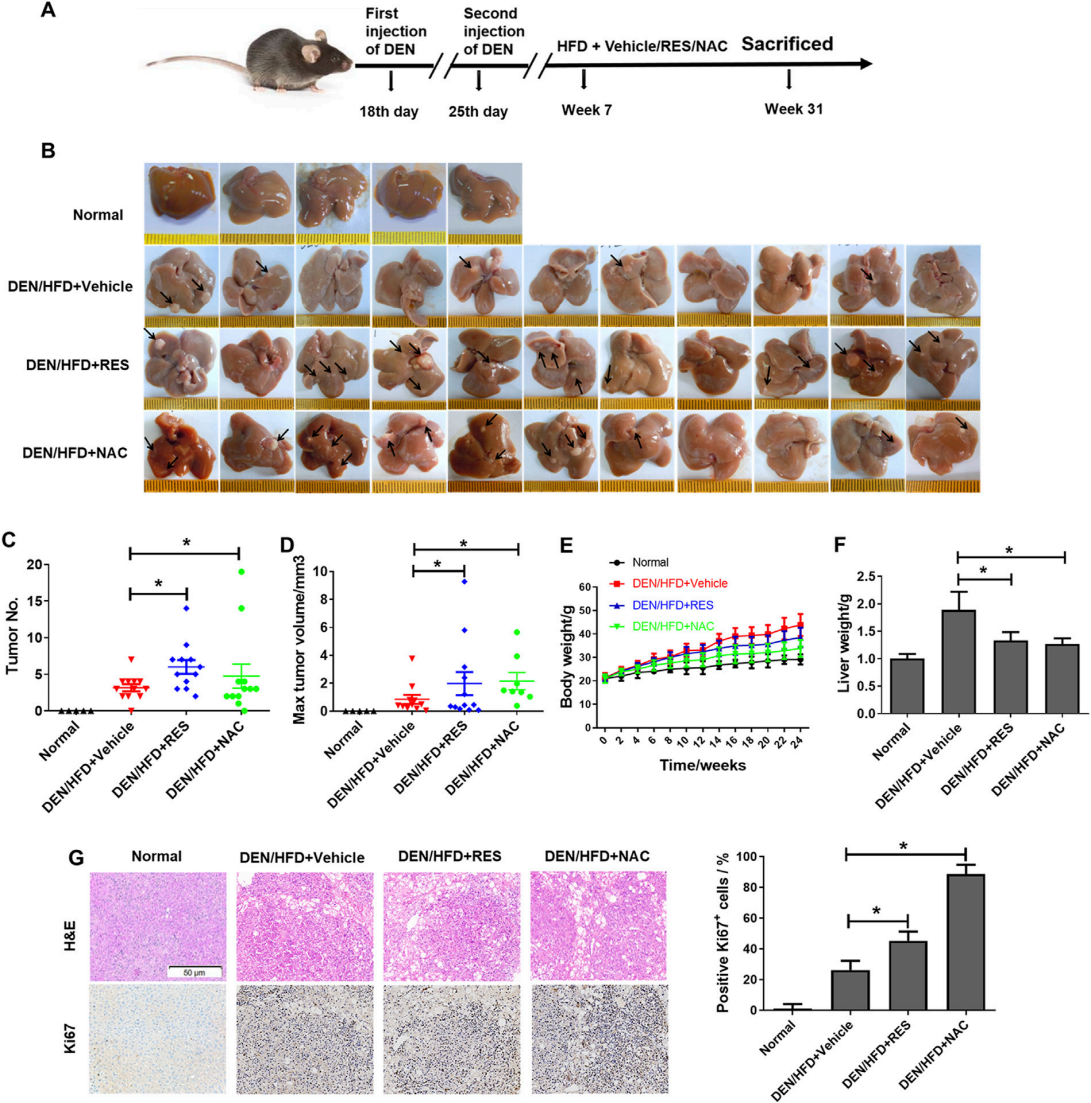
RES and NAC enhance HCC formation in DEN/HFD mice. **(A)** DEN/HFD-induced HCC development in mice and the experimental design. **(B)** Representative images of the liver of mice treated with vehicle, 0.4% w/w RES, or 1 g/L NAC. Black arrows indicate HCC nodules. **(C–F)** Tumor number **(C)**, maximum tumor volume **(D)**, body weight **(E)**, and liver weight **(F)** of each group. **(G)** H&E and IHC staining of mouse HCC tissues, and the percentages of Ki67-positive cells. Scale bar = 50 μm. Data are presented as mean ± SD (n = 11), with statistical significance denoted as *P < 0.05.

**FIGURE 2:**
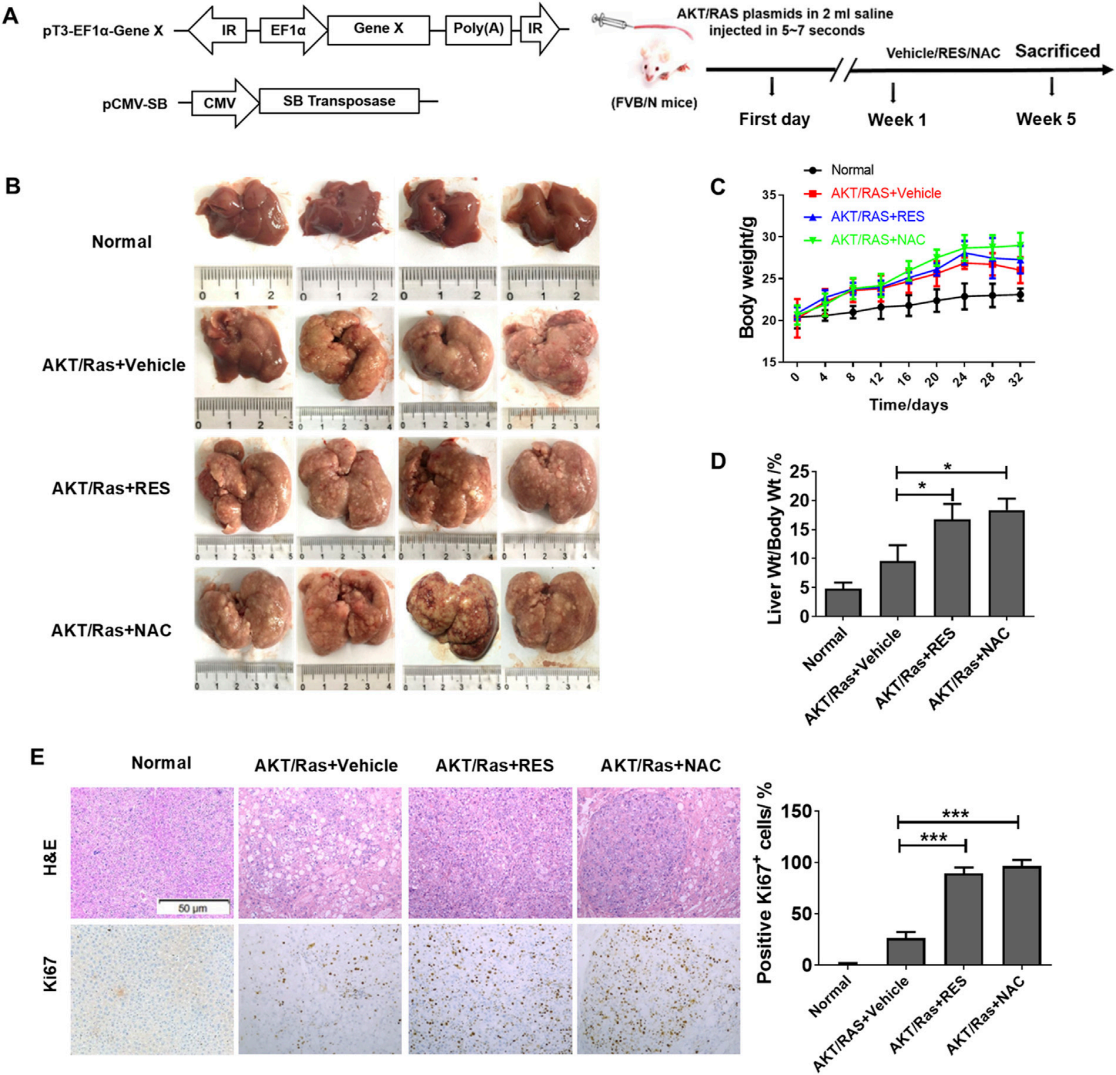
RES and NAC enhance HCC in AKT/Ras mice. **(A)** AKT/Ras-induced HCC development and the experimental design. **(B)** Representative images of the liver of AKT/Ras mice treated with vehicle, 0.4% RES, or 1 g/L NAC. **(C)** Body weight gain during tumor development. **(D)** Liver-to-body weight ratio. **(E)** H&E and IHC staining of mouse HCC tissues, and the percentages of Ki67-positive cells. Scale bar = 50 μm. Data are presented as mean ± SD (n = 4), with statistical significance denoted as *P < 0.05, **P < 0.01, ***P < 0.001.

To evaluate the effects of antioxidants on HCC formation during steatosis, we administered RES and NAC to DEN/HFD and AKT/Ras mice, respectively. DEN/HFD mice serve as a steatosis- and carcinogen-induced HCC model, and AKT/Ras mice represent a steatosis- and oncogene-induced HCC model because AKT drives lipogenesis in the mouse liver. RES and NAC were chosen for two reasons. First, these two antioxidants have been reported to alleviate mouse hepatosteatosis (Ma et al., 2016; Jeon et al., 2012). Second, RES and NAC are different types of antioxidants. RES is fat-soluble, functions as a superoxide and metal-induced radical scavenger (Neves et al., 2012; Leonard et al., 2003) and exerts caloric restriction effect (Tennen et al., 2012). NAC is water-soluble and participates in GSH metabolism (Sadowska, 2012; Samuni et al., 2013). DEN/HFD mice were fed vehicle, 0.4% w/w RES, or 1 g/L NAC diet for 24 weeks after DEN injection (Figure 1A). RES was mixed with HFD chow at 0.4% w/w. NAC was administered to mice in drinking water at a concentration of 1 g/L. At the end of the experiment, vehicle-treated mice had an average of four nodules on the liver surface (Figures 1B,C). In contrast, RES- and NAC-treated mice had an average of six and seven nodules, respectively. The average maximal tumor volume in vehicle-treated mice was 1 mm^3^, whereas that in RES- and NAC-treated mice were 2 and 3 mm^3^, respectively (Figure 1D). RES and NAC led to 2.1- and 1.7-fold increased tumor burden compared with the vehicle group, respectively. Interestingly, both RES and NAC reduced the body and liver weights of mice fed HFD since the fourth week (Figures 1E,F). Ki67 staining showed that RES and NAC increased proliferation in the liver of treated mice (Figure 1G). AKT/Ras mice were fed vehicle, 0.4% w/w RES, or 1 g/L NAC diet for 5 weeks after AKT/Ras plasmid injection (Figure 2A). RES was mixed with HFD chow at 0.4% w/w. NAC was administered to the mice in drinking water at a concentration of 1 g/L. Mouse liver was found covered with tumor nodules at the fifth week after plasmid injection (Figure 2B). RES or NAC administration significantly increased the body weight and liver/body weight ratio of mice, indicating that both agents increased HCC formation in AKT/Ras mice (Figures 2C,D). Similar to that in DEN/HFD mice, RES or NAC treatment increased the proliferation of HCC cells in AKT/Ras mice (Figure 2E). These results indicated that both RES and NAC enhanced HCC initiation in mice with steatosis.

### 3.2 RES and NAC alleviate hepatosteatosis in mice

**FIGURE 3:**
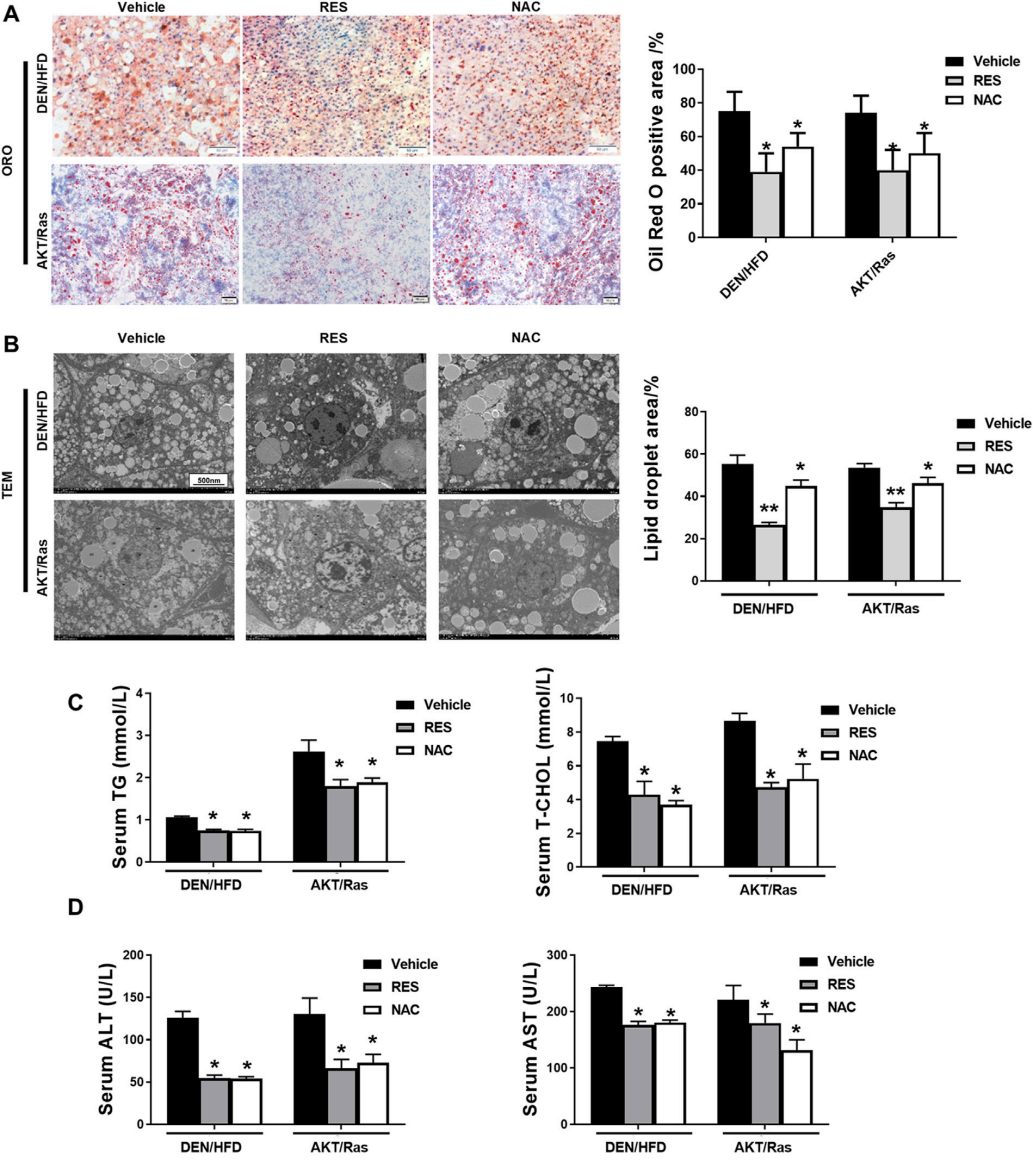
RES and NAC reduce lipid accumulation in DEN/HFD- and AKT/Ras-induced HCC mice. **(A)** Representative ORO staining of mouse liver sections, and the percentage of ORO-positive area. Scale bar = 50 μm. **(B)** TEM images of lipid droplets in mouse liver slices, and the percentage of lipid droplet area. Scale bar = 500 nm. **(C)** Serum levels of TG and TC in HCC mice. **(D)** Serum levels of ALT and AST in HCC mice. The data are presented as mean ± SD (n = 4). Statistical significance is denoted as *P < 0.05, **P < 0.01.

In both DEN/HFD and AKT/Ras mice, HCC is partially attributed to lipogenesis or lipid accumulation causing liver damage (Ho et al., 2012; Park et al., 2010). In addition, we observed that fatty liver or lipoma formed earlier than HCC in the DEN/HFD and AKT/Ras mouse HCC models (Supplementary Figure S1). To examine whether RES and NAC accelerate HCC by regulating lipid metabolism, we performed ORO staining and transmission electron microscopy (TEM). The result showed that both RES and NAC treatment led to smaller cytoplasmic lipid droplets in mouse hepatocytes than those in the vehicle group (Figures 3A,B). Consistent with the reduced ORO staining, RES and NAC treatment reduced the serum levels of TG and TC (Figure 3C). Furthermore, serum ALT and AST levels decreased after RES or NAC treatment (Figure 3D). This implied that RES and NAC mitigated lipid accumulation in mice with HCC induced by both DEN/HFD and AKT/Ras, although they accelerated HCC development.

### 3.3 Antioxidants reduce ROS and DNA damage and increase tumor cell proliferation *in vivo*

**FIGURE 4:**
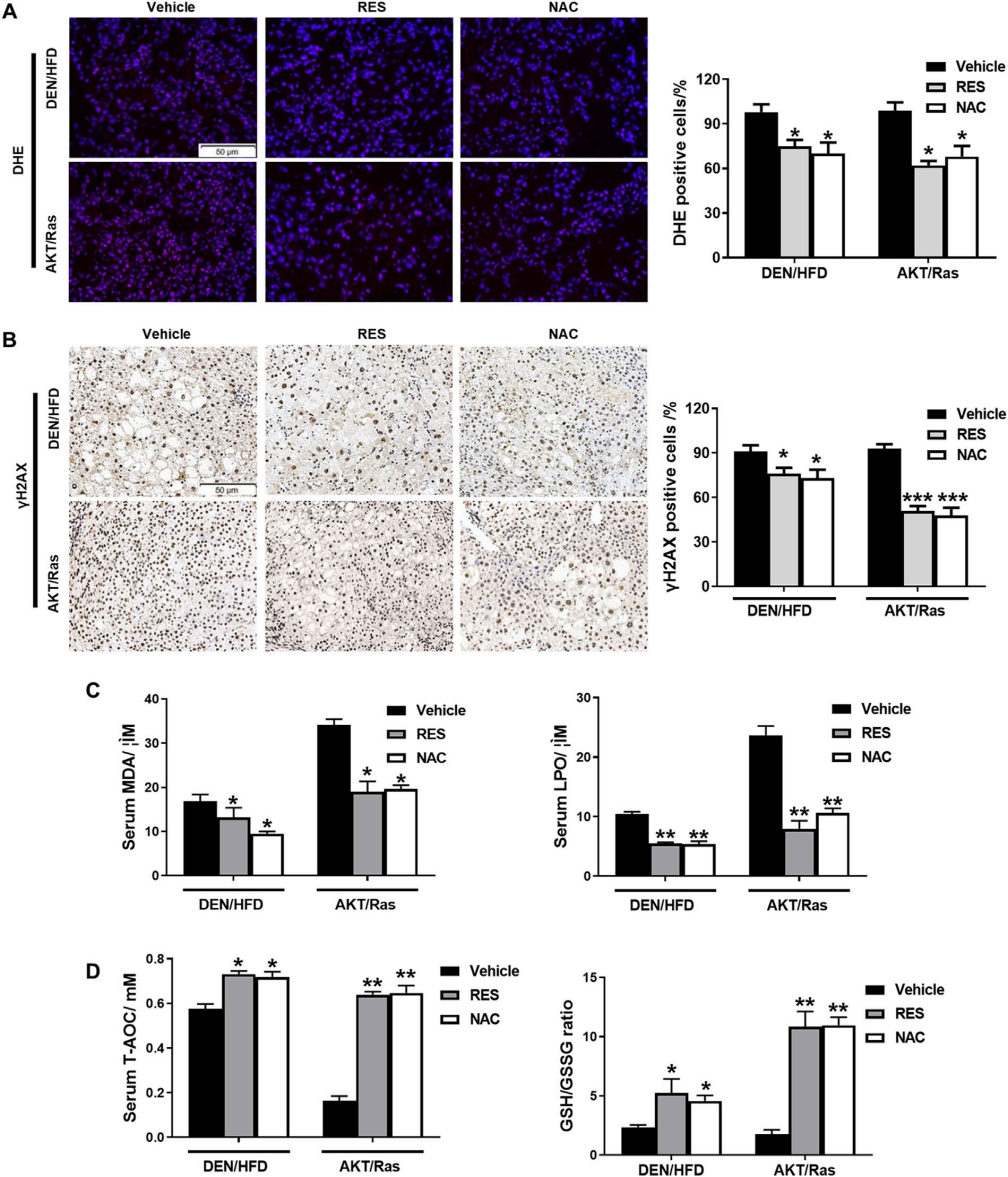
RES and NAC reduce ROS accumulation, DNA damage, and lipid peroxidation in DEN/HFD- and AKT/Ras-induced HCC mice. **(A)** ROS detection using DHE staining in HCC mouse liver sections and the percentage of DHE-positive cells. Scale bar = 50 μm. **(B)** γH2AX staining of HCC mouse liver sections, and the percentages of γH2AX-positive cells. Scale bar = 50 μm. **(C)** Serum MDA and LPO levels in HCC mice. **(D)** Serum total antioxidant capacity (T-AOC) and GSH/GSSG ratio in HCC mice. Data are presented as mean ± SD (n = 4); *P < 0.05, **P < 0.01.

Studies have shown that NAC and vitamin E reduce ROS and DNA damage in lung tumor cells, thereby facilitating the proliferation of tumor cells (Sayin et al., 2014). NAC and Trolox alleviated DNA damage in DEN-treated primary hepatocytes (Wang B. B. et al., 2018). Thus, we examined ROS levels and DNA damage responses in the liver tissues of RES- and NAC-treated mice. Staining with the redox-sensitive probe DHE showed that ROS levels in HCC mouse tissues were reduced by RES and NAC (Figure 4A). Immunohistochemistry (IHC) analyses of γH2AX indicated that RES and NAC alleviated ROS-induced DNA damage as well (Figure 4B). We subsequently investigated the effect of antioxidants on lipid peroxidation activity and found that antioxidant treatment significantly reduced MDA and LPO activities in the serum of mice compared to those in untreated model group mice (Figure 4C). In contrast, antioxidant treatment increased the levels of T-AOC and GSH/GSSG in the serum of mice compared to those in untreated HCC mice (Figure 4D). Taken together, these data indicate that antioxidants reduce ROS and DNA damage, although they accelerate tumor formation or growth *in vivo*.

### 3.4 Antioxidants increase the expression of GST-pi in HCC

**FIGURE 5:**
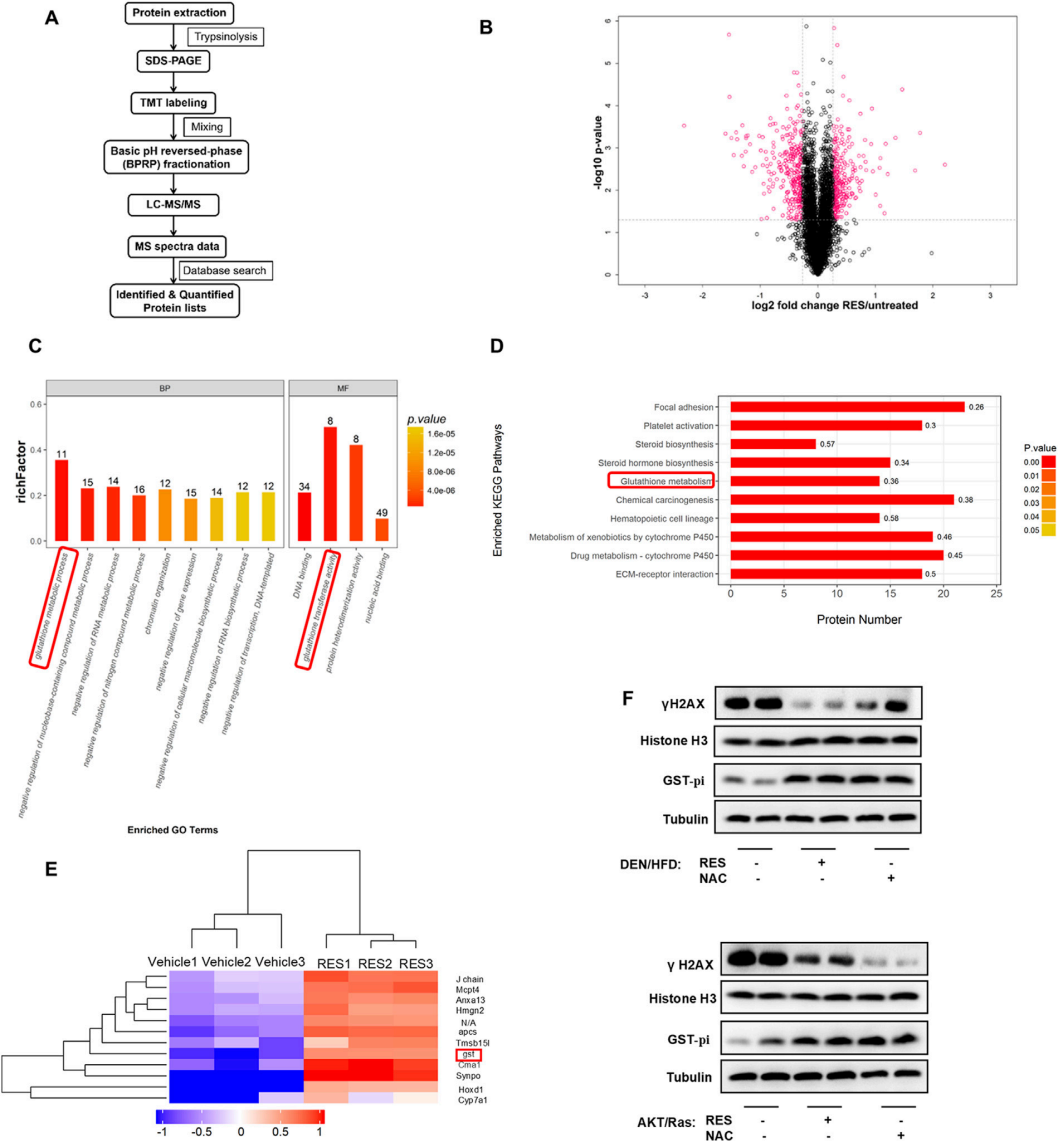
Proteomics screened GST-Pi as a potential effector upon antioxidants treatment. **(A)** Proteomic screening procedure is illustrated. **(B)** Volcano plot of average peptide intensities in both RES- and vehicle-treated HCC tissues. The horizontal dotted line indicates *P* < 0.05. Proteins with a fold change > 1.2 are shown in pink (n = 3). **(C)** GO enrichment analysis of the upregulated proteins using Fisher’s exact test. The top nine enriched biological pathways and top four molecular function pathways are shown (n = 3). **(D)** The top 10 KEGG pathways enriched in RES-treated mouse HCC tissues (n = 3). **(E)** The top 12 hits with the highest increase were selected for clustering analysis. The colors indicate relative abundance; red indicates higher abundance and blue indicates lower abundance (n = 3). **(F)** Western blot analysis of γH2AX and GST-pi in mouse liver tissues treated with antioxidants. Histone H3 was used as the internal reference for nucleoproteins. Tubulin was used as the loading control.

We investigated how antioxidants enhance HCC growth while blocking ROS production and DNA damage. To this end, we performed longitudinal, unbiased, quantitative proteomics using liver tissues from RES-treated and vehicle-treated DEN/HFD mice (Figure 5A). A total of 4,927 proteins were detected, of which 551 showed significant (>2-fold change) upregulation or downregulation in the liver tissues of RES-treated mice compared to that of vehicle-treated mice (Figure 5B). Pathway enrichment analysis revealed enrichment of the GSH metabolic pathway (Figure 5C). Consistently, KEGG analysis showed that the GSH metabolic pathway was enriched (Figure 5D). We screened differentially expressed proteins between the vehicle-treated and RES-treated groups and found that GST showed 2.76-fold higher expression in the RES-treated group than in the vehicle-treated group (Figure 5E). GSTs are a group of phase II detoxification enzymes that facilitate the binding of GSH to different types of endogenous and exogenous electrophilic molecules. The cytoplasmic GST superfamily consists of at least seven classes, with the most abundant in mammals being the alpha, mu, and pi classes of enzymes (Hayes and Pulford, 1995; Townsend and Tew, 2003). Recent studies have shown that GST-pi is highly expressed in liver tumor cells (Su et al., 2003) and is closely related to carcinogenesis, tumor formation (Dang et al., 2005) and the annual survival rate of various tumors (Yasuno et al., 1999). We therefore performed Western blot to detect GST-pi, and found that GST-pi expression was increased in the liver tissues of both the RES- or NAC-treated mice, whereas nucleoprotein γH2AX was decreased (Figure 5F). Taken together, these results indicate that GST-pi is involved in the promotion of HCC growth by antioxidants.

### 3.5 RES and NAC accelerate HCC initiation and progression via GST-pi

**FIGURE 6:**
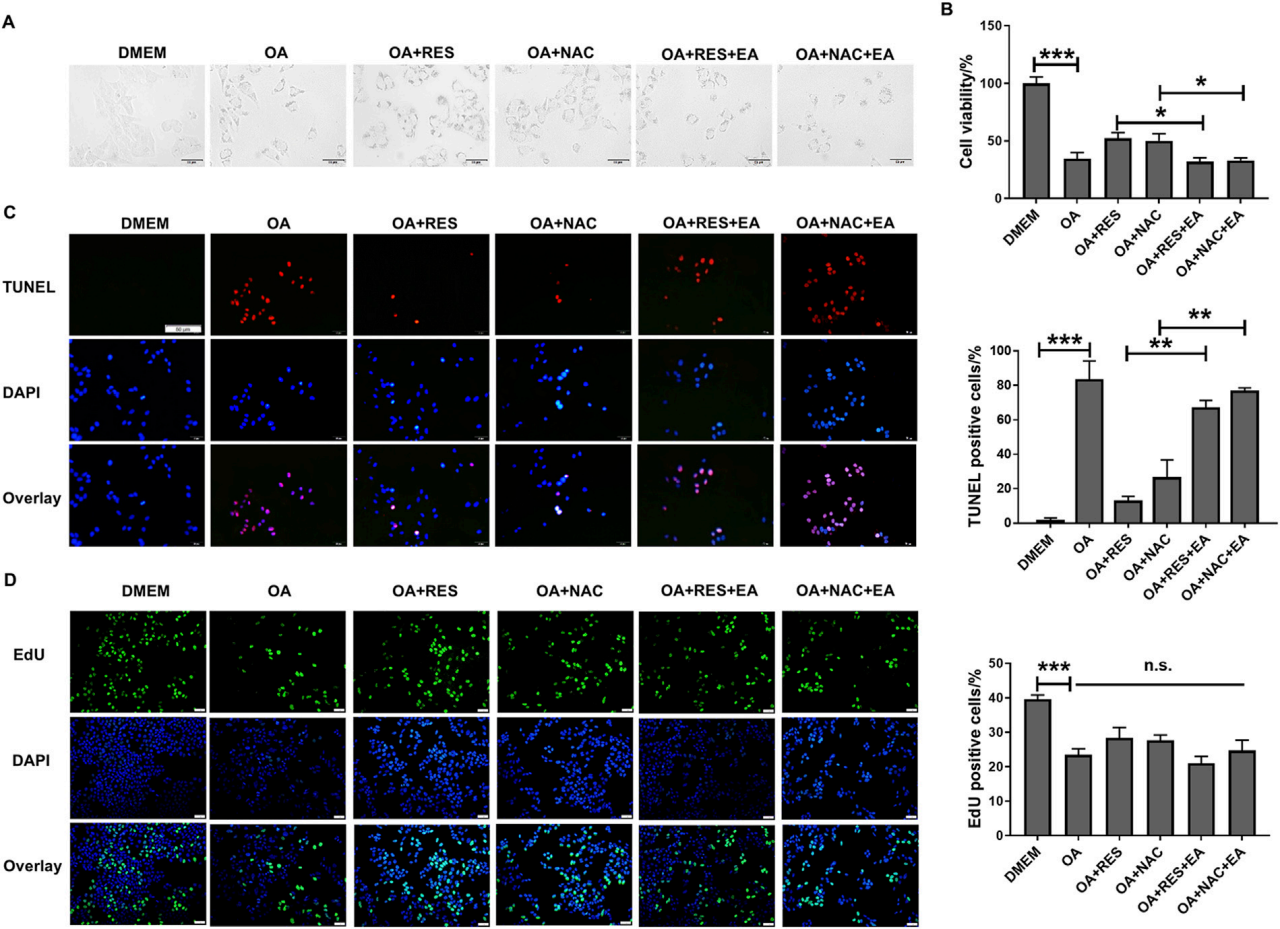
RES and NAC inhibit HCC cell apoptosis but do not accelerate cell proliferation *in vitro*. **(A)** Morphology of HepG2 cells. HepG2 cells treated with OA (0.8 mM) for 24 h with and without 25 μM RES or 250 μM NAC or/and 20 μM EA. **(B)** The viability of HepG2 cells was determined using the CCK-8 assay. **(C)** TUNEL staining of HepG2 cells, and the percentage of TUNEL-positive cells. Scale bar = 50 μm. **(D)** EdU staining of HepG2 cells, and the percentage of EdU-positive cells. Scale bar = 50 μm. Data are presented as mean ± SD (n = 3), n.s., not significant; *P < 0.05, **P < 0.01, ***P < 0.005.

GST-pi plays a critical role in promoting tumorigenesis and drug resistance of tumor cells (Crawford and Weerapana, 2016). Next, we tested whether RES and NAC accelerated tumor progression through the activation of GST-pi. Since the primary hepatocytes can only be cultured for a short time and cannot grow *in vitro*, we chose HCC cell lines to evaluate the roles of RES and NAC in HCC development *in vitro*. We treated HepG2 cells with 0.8 mM OA and subsequently with 25 μM RES or 250 μM NAC. We found that RES or NAC significantly restored cell morphology (Figure 6A), increased cell viability (Figure 6B), and reduced apoptosis caused by OA treatment (Figure 6C), but did not restore proliferation at a series of concentrations (Figure 6D; Supplementary Figure S2). We then treated HepG2 cells with 20 μM EA, a well-characterized GST family inhibitor (Hansson et al., 1991). Following concomitant EA treatment, RES and NAC failed to reduce apoptosis in HepG2 cells. These results suggested that the promoting effect of antioxidants on HCC was dependent on GST-pi *in vitro*.

**FIGURE 7:**
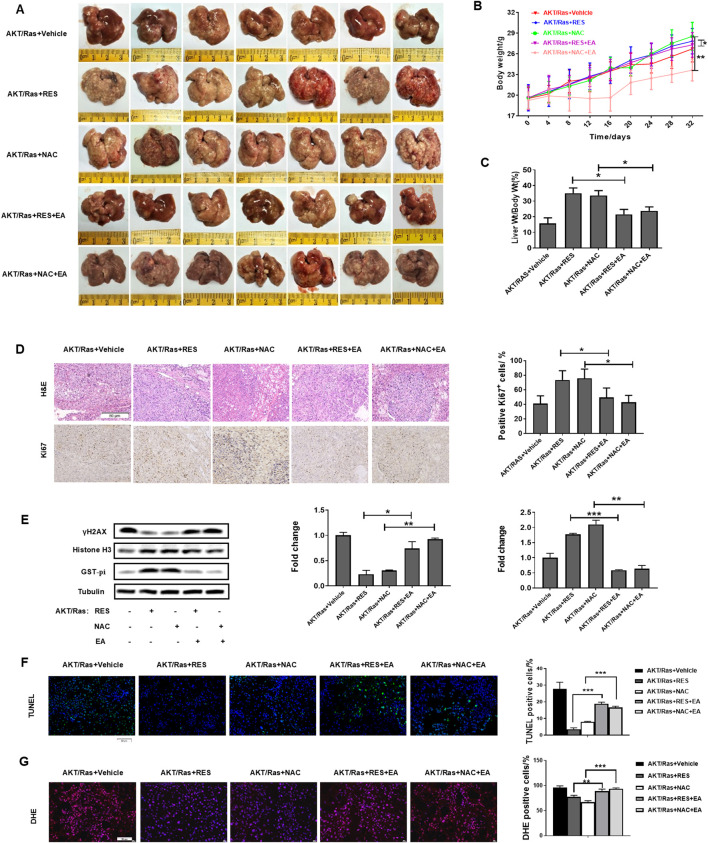
GST-pi inhibitor abrogates the promoting effects of antioxidants on HCC progression. AKT/Ras mice were treated with vehicle, 0.4% w/w RES, 0.4% w/w RES + 25 mg/kg/day EA, 1 g/L NAC, or 1 g/L NAC + 25 mg/kg/day EA. **(A)** Liver morphology of mice (n = 7). **(B)** Body weight gain during tumor development. **(C)** Liver-to-body ratio (n = 7). **(D)** HE or IHC staining showing HCC histology and AKT, Ras, and Ki67 expression. Scale bar = 50 μm (n = 7). **(E)** Western blot of γH2AX and GST-pi in the liver tissues (n = 2). Histone H3 was used as the internal reference for nucleoproteins. Tubulin was used as the loading control. **(F)** TUNEL staining of HCC mouse liver sections, and the percentage of TUNEL-positive cells. Scale bar = 50 μm (n = 7). **(G)** ROS detection by DHE staining of HCC mouse liver sections. Scale bar = 50 μm. Data are presented as mean ± SD (n = 7); *P < 0.05, **P < 0.01, ***P < 0.005.

We explored the effects of EA administration on the growth of AKT/Ras-induced HCC cells treated with RES or NAC. Treatment with 25 mg/kg/day EA abrogated the promoting effects of 0.4% w/w RES and 1 g/L NAC on HCC progression (Figure 7A). EA treatment also reduced the body weight and liver/body weight ratio of mice (Figures 7B,C). Moreover, EA treatment decreased tumor cell proliferation and increased DNA damage, apoptosis, and ROS levels (Figures 7D–G). Collectively, these results indicate that GST-pi mediates the promoting effect of antioxidants in HCC initiation and progression.

### 3.6 RES and NAC enhance tumor growth by inhibiting MAPK pathway

**FIGURE 8:**
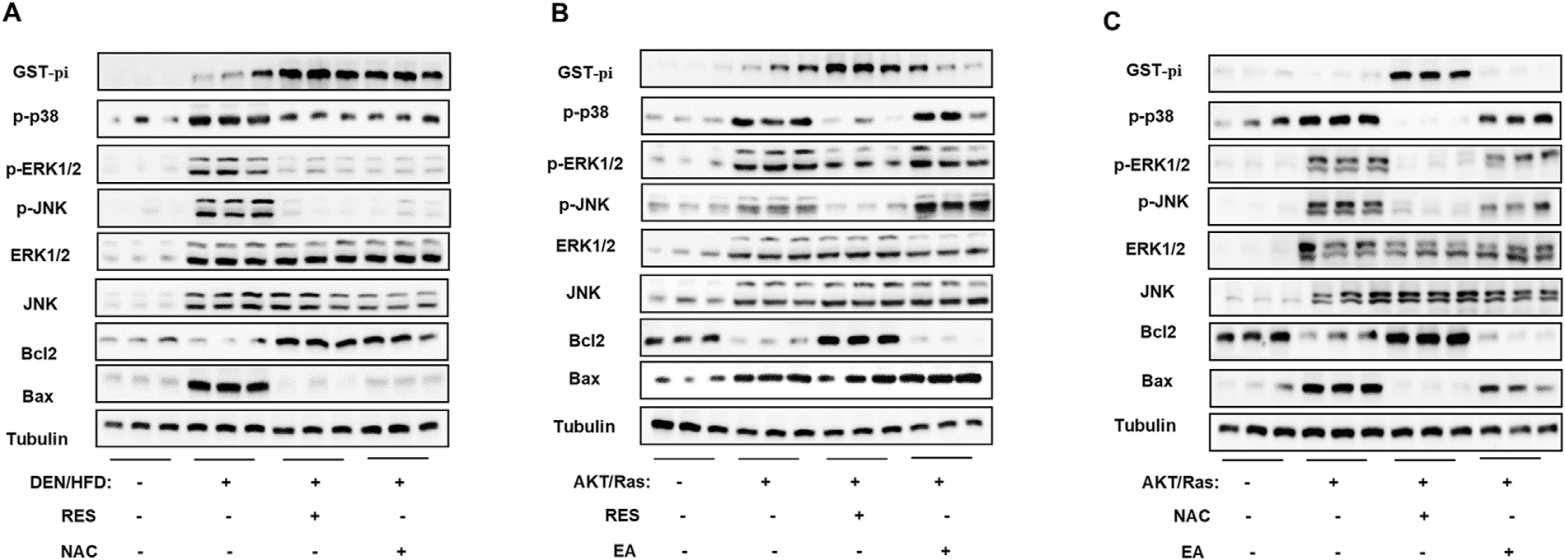
RES and NAC inhibit the MAPK pathway through GST-pi. **(A)** Western blotting of GST-pi, p-p38, p-ERK1/2, ERK1/2, p-JNK, JNK, Bcl2, and Bax in the liver tissues of DEN/HFD model mice fed HFD + vehicle, HFD + RES, or HFD + NAC. **(B, C)** Western blotting of proteins in the liver tissues of AKT/Ras mice fed normal chow plus EA plus RES **(B)** or NAC **(C)**, respectively.

Next, we investigated the mechanism by which GST-pi accelerates HCC progression. Recent studies have reported that GST-pi regulates the mitogen-activated protein kinase (MAPK) pathway, which is frequently activated in HCC (Sciskalska and Milnerowicz, 2020; Moon and Ro, 2021). To date, six groups of MAPKs have been identified, of which ERK1/2, JNK, and p38 are the three major ones (Sui et al., 2014). Thus, we assessed the activation of ERK1/2, JNK, and p38, and found that the ERK1/2, JNK, and p38 pathways were all inhibited, along with increased GST-pi in DEN/HFD-induced HCC treated with RES or NAC (Figure 8A). This result is consistent with the reported regulation of p38 (Sciskalska and Milnerowicz, 2020), ERK1/2 (Wang et al., 2019), and JNK by GST-pi (Thevenin et al., 2011; Townsend and Tew, 2003). In addition, in AKT/Ras-induced HCC model, antioxidants increased GST-pi protein levels and inhibited the activation of MAPK pathway proteins. EA significantly reduced GST-pi protein levels and activated MAPK pathway proteins (Figures 8B,C). The MAPK pathway is an important signaling pathway that promotes apoptosis (Sui et al., 2014; Wang J. et al., 2018). As expected, the expression of the anti-apoptotic protein Bcl2 increased and that of the pro-apoptotic protein Bax decreased upon RES or NAC treatment. Conversely, Bcl2 was inhibited, whereas Bax increased in the presence of the GST-pi inhibitor. Taken together, these results indicate that antioxidants inhibit apoptosis via the GST-pi-MAPK axis and play tumor-promoting role in HCC.

## 4 Discussion

Antioxidants, including vitamins, carotenes, and minerals, have long been used for cancer prevention and treatment because of their ability to neutralize ROS (Willcox et al., 2004; Giorgio, 2015). However, the antitumor effects of antioxidants have never been validated in clinical trials. Accumulating evidence has confirmed that antioxidants may accelerate tumor growth and metastasis. However, whether antioxidants can be used as daily supplements for prevention has not been fully addressed at both the whole-body and molecular levels. In this study, we demonstrated that RES and NAC enhanced HCC formation in both DEN/HFD- and AKT/Ras-induced HCC mouse models. In terms of mechanism, RES and NAC were found to upregulate GST-pi expression, inhibit the MAPK pathway, and reduce ROS, DNA damage, and apoptosis of HCC cells.

The promoting effect of antioxidants on HCC progression in our study is consistent with the results obtained in other tumor models in previous studies. In mouse lung cancer, NAC and vitamin E increased cell proliferation by reducing ROS and DNA damage and disrupting the ROS-p53 axis (Sayin et al., 2014). Similarly, in human malignant melanoma cells, NAC and the soluble vitamin E analog Trolox enhanced migration and invasion without affecting cell proliferation. In contrast, in DEN-induced HCC mouse model, the non-mitochondria-targeting antioxidants NAC and Trolox prevented tumorigenesis, whereas the mitochondria-targeting antioxidants Mito-Q and SS-31 promoted tumorigenesis (Wang B. B. et al., 2018). Together with our study, these findings suggest that patients with HCC should avoid taking antioxidants or use antioxidant supplements with caution. However, whether the data obtained from mice can be generalized to humans needs to be confirmed in clinical trials or cohort studies.

Fat accumulation was reportedly correlated with increased ROS in humans and mice, and increased ROS levels promote cell proliferation and differentiation (Boonstra and Post, 2004; Schafer and Buettner, 2001; Furukawa et al., 2004). Moreover, the risk of developing HCC can be further increased through the synergistic effects of HCV infection and NAFLD (Li et al., 2009; Parekh and Anania, 2007). Therefore, people may hold that antioxidants may delay or prevent cancer initiation in patients with NAFLD. However, our results indicate that RES and NAC can enhance tumor formation and development in mice, even with a high-fat diet or lipogenesis. Indeed, inhibition of hepatic lipogenesis in mice treated with DEN increased tumor incidence (Nelson et al., 2017). Inhibiting lipogenesis results in a significant increase in the levels of endogenous antioxidants such as NADPH and reduced GSH. This finding supports our conclusion that liver tumorigenesis in mice treated with DEN or driver oncogenes does not depend on lipogenesis. Our results suggest that NAC and RES enhance the initiation and development of HCC in mice. However, further investigations are needed to determine whether all antioxidants that can reduce lipid accumulation or inhibit hepatic steatosis, such as vitamin C, vitamin E (Shin, 2003), and puerarin flavonoids (Sun et al., 2023), can enhance the development of HCC. Whether all antioxidants enhance HCC formation requires further investigation, particularly because some antioxidants do not reduce lipid accumulation or inhibit hepatic steatosis. Therefore, their roles in blocking HCC formation and progression in the context of fatty liver warrant further investigation.

Our study highlighted the pivotal role of GST-pi in ROS scavenging and HCC. It has been reported that GST-pi is upregulated in pre-neoplastic lesions observed in animal cancer models induced by chemicals (Satoh et al., 1985) and a wide range of human tumors (Shea et al., 1988). When colon cancer cells are cultured under growth-limiting conditions, a deficiency in GST-pi expression leads to increased cellular oxidative stress, leading to apoptosis (Dang et al., 2005). Subsequent *in vivo* experiments showed that GST-pi had a significant impact on the initial stages of cancer development. In breast cancer, tumors that express GST-pi have been shown to be more aggressive and had worse prognosis compared to tumors that do not express GST-pi (Huang et al., 2003). GST-pi expression was found in 62.4% of ovarian tumors and directly affected the chemosensitivity of ovarian tumor cell lines to platinum drugs (Ikeda et al., 2003; Sawers et al., 2014). The pivotal role of GST-pi in carcinogenesis may depend on the regulation of several critical kinases in cancer cells. When exposed to chemical or oxidative stress, the dissociation of the GST-pi-JNK complex led to the release of GST-pi for oligomerization. Released JNK was then activated, triggering apoptosis (Adler et al., 1999; Davies et al., 2001). MAPK kinase and ERK1/2 kinase signaling in colon cancer was dependent on the presence of GST-pi, in line with our observations in this study (Dang et al., 2005).

Our findings provide further evidence supporting the controversial role of MAPK in HCC. ERK1/2, JNK, and p38 are the three major MAPK proteins (Sui et al., 2014). ERK1/2 is mainly activated by growth signals and promotes HCC cell growth (Moon and Ro, 2021), while JNK and p38 are mainly activated by environmental stress and play dual roles in various cancers (Sui et al., 2014). Inhibition of ERK can lead to apoptosis, whereas the inhibition of JNK and p38 can prevent apoptosis (Huynh et al., 2003). In this study, we found that RES and NAC increased GST-pi expression and subsequently inhibited the activation of JNK and p38, suggesting that JNK and p38 promoted cell apoptosis in HCC. In addition, JNK and p38 are capable of balancing autophagy and apoptosis (Sui et al., 2014). Whether autophagy is involved in the pro-tumoral role of antioxidants merits further investigation.

Notably, the conclusion that NAC and RES enhance HCC incidence only applies to the liver with aberrant lipid metabolism. Whether NAC and RES accelerate HCC along with other risk factors remains controversial. Several studies have shown that NAC and RES inhibit DEN-induced HCC and that elevated ROS level is required for tumor development (Bishayee et al., 2010; Lin et al., 2013). However, other studies have demonstrated that NAC promotes tumor growth (Sayin et al., 2014; Schafer et al., 2009). A prospective cohort study conducted from 1998 to 2009 showed that total urinary RES metabolite concentration was not associated with cancer mortality (Semba et al., 2014). In addition, *in vitro* experiments demonstrated that RES and NAC reduced the apoptosis of HCC cells pre-treated with OA but did not enhance cell proliferation, which was not fully consistent with the *in vivo* results. These results suggest that the stimulatory effects of antioxidants on HCC growth depend on the tumor microenvironment (TME). HCC TME contains many stromal and immune cell types, including fibroblasts, endothelial cells (Wang et al., 2022), regulatory T cells (Zhang et al., 2022), myeloid-derived suppressor cells (Hoechst et al., 2009), neutrophils (Geh et al., 2023), and tumor-associated macrophages (TAM) (Cheng et al., 2022). For instance, TAM can be repolarized by antioxidants to M1 in bladder cancer, enhancing the efficacy of anti-PD-L1 immunotherapy (Ma et al., 2022). Therefore, we speculate that antioxidants may also enhance HCC by directly regulating the TME, in addition to their roles in HCC cells, and we will further investigate these effects in future studies.

## Data Availability

The mass spectrometry proteomics data have been deposited to the ProteomeXchange Consortium (https://proteomecentral.proteomexchange.org) via the iProX partner repository with the dataset identifier PXD056132.
