# Supplementary material for: Resveratrol and N-acetylcystein reduce hepatic steatosis but enhance initiation and progression of hepatocellular carcinoma by inhibiting GST-pi-MAPK axis in mice
Source: Front Pharmacol. 2025 Apr 28;16:1574039. doi: 10.3389/fphar.2025.1574039 (PMC12066552; doi:10.3389/fphar.2025.1574039)

# Original blots and images for explanation

## Original blots for Figure 5F


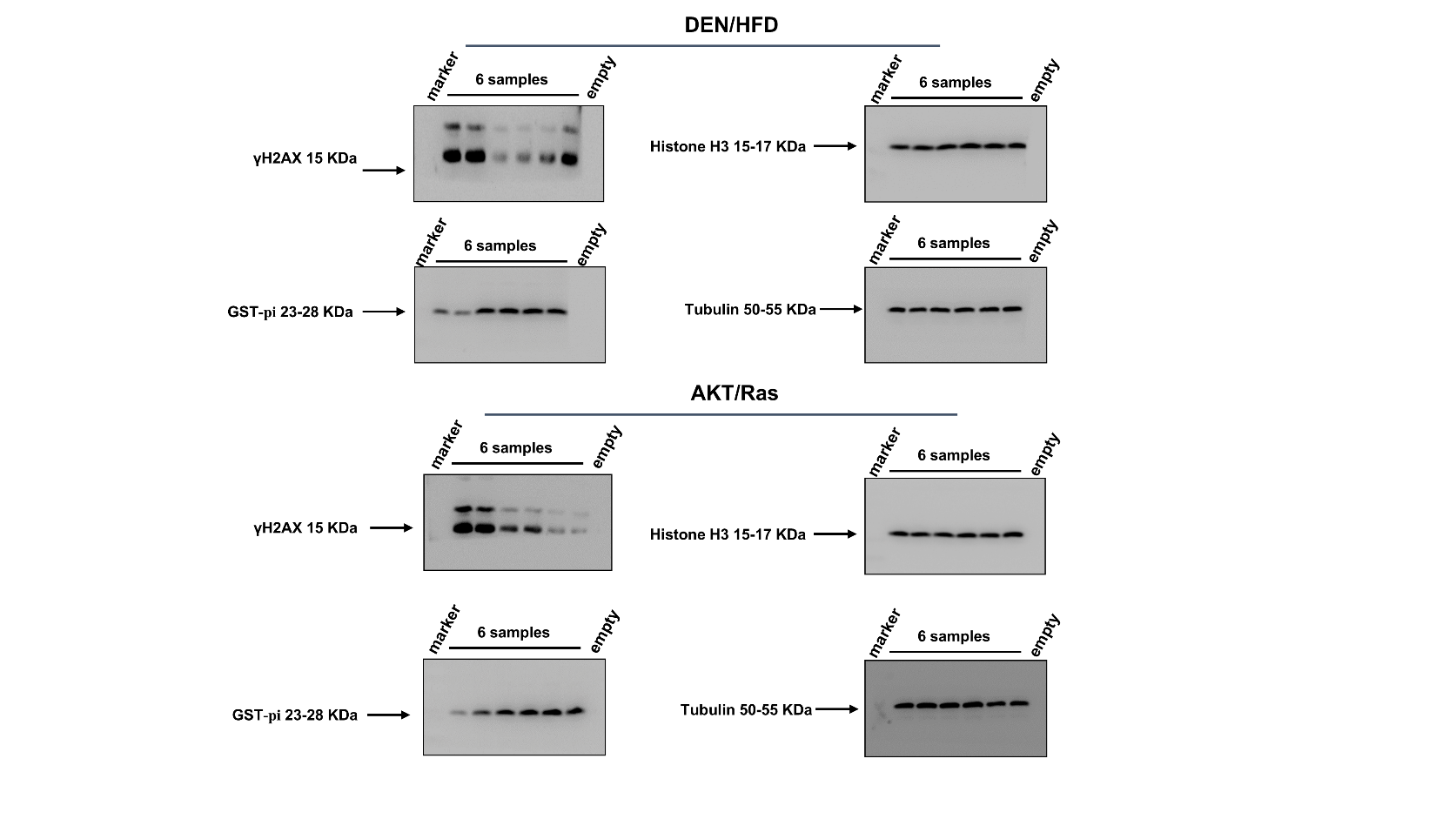


## Original blots for Figure 7E


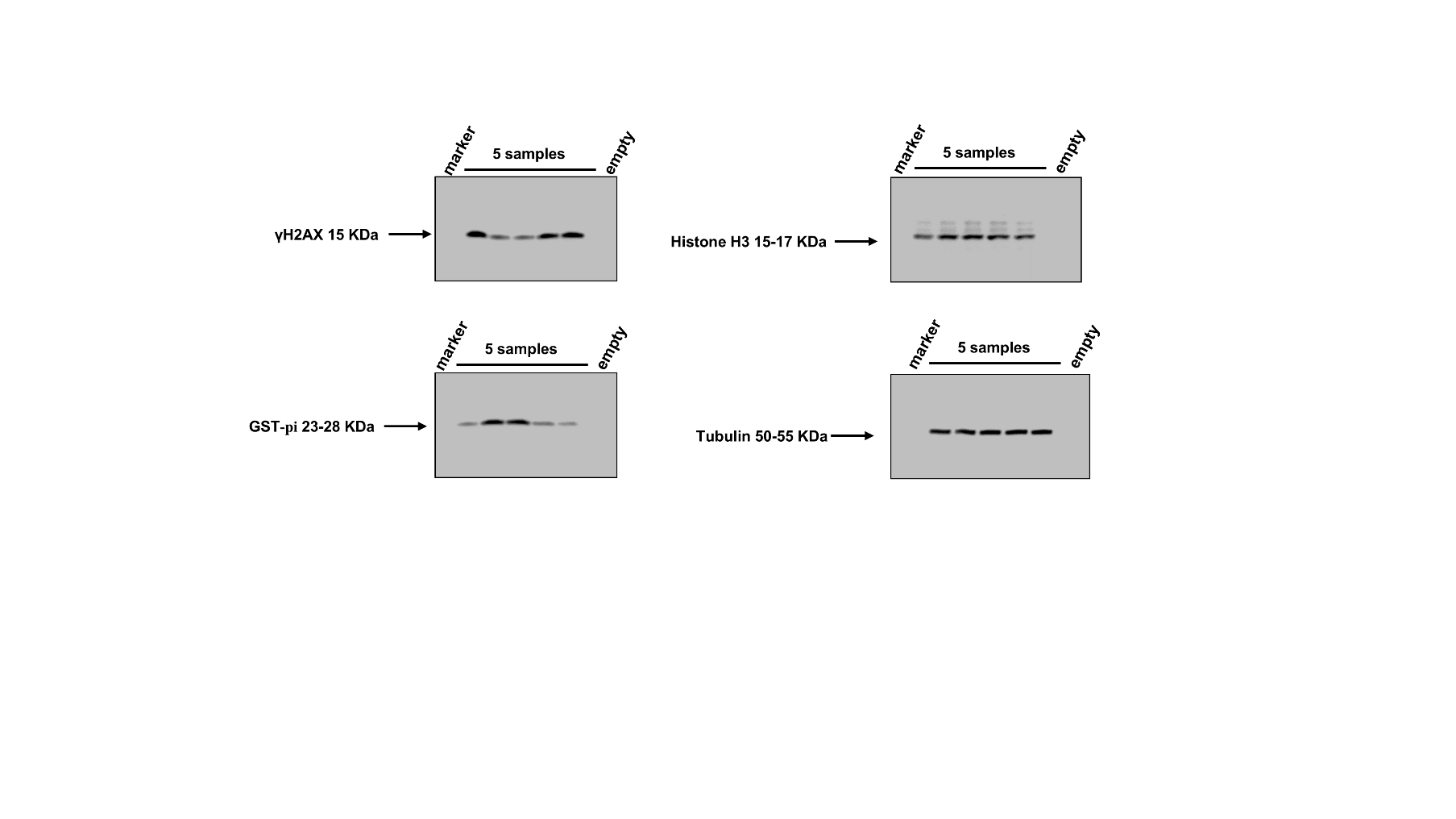


## Original blots for Figure 8A


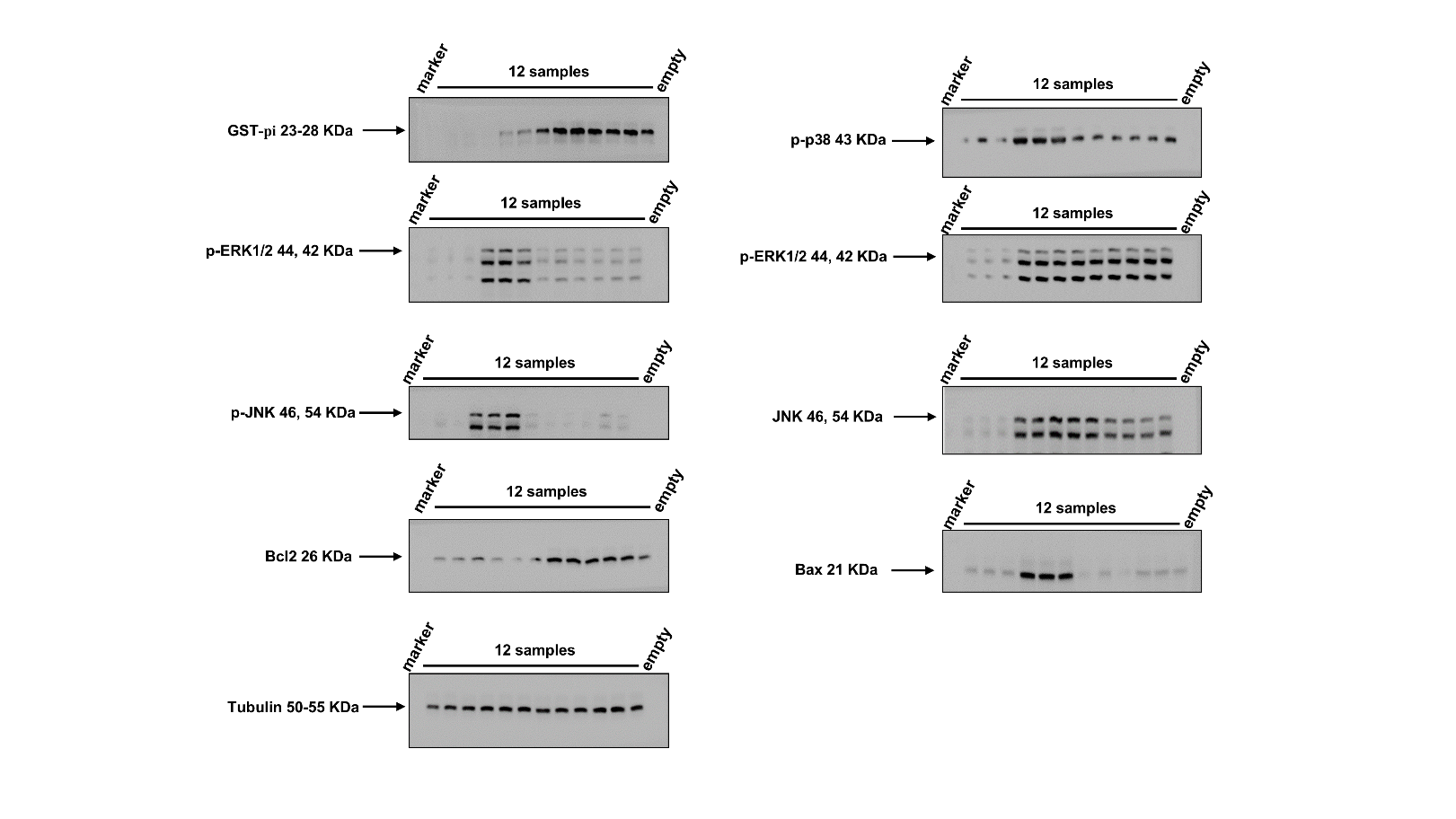


## Original blots for Figure 8B


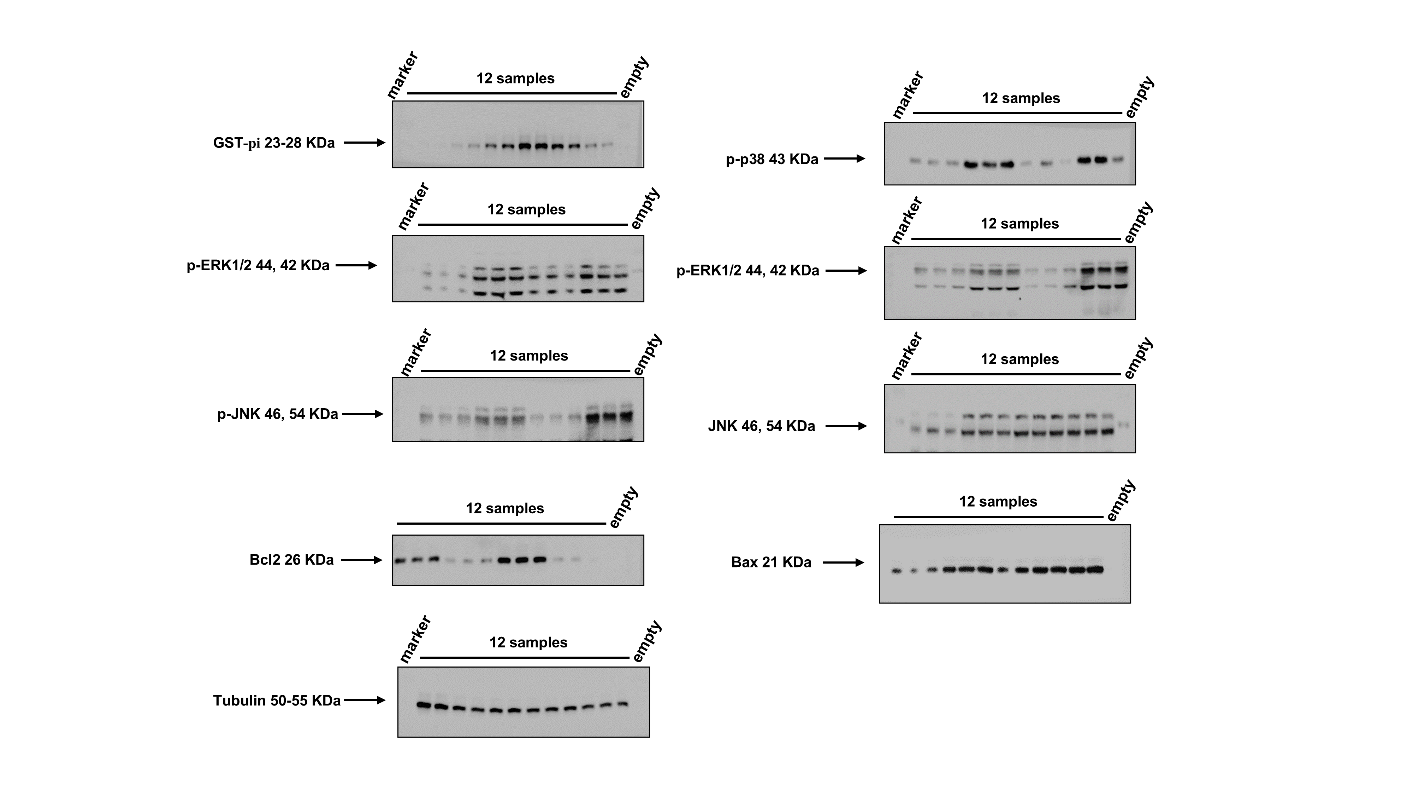


## Original blots for Figure 8C


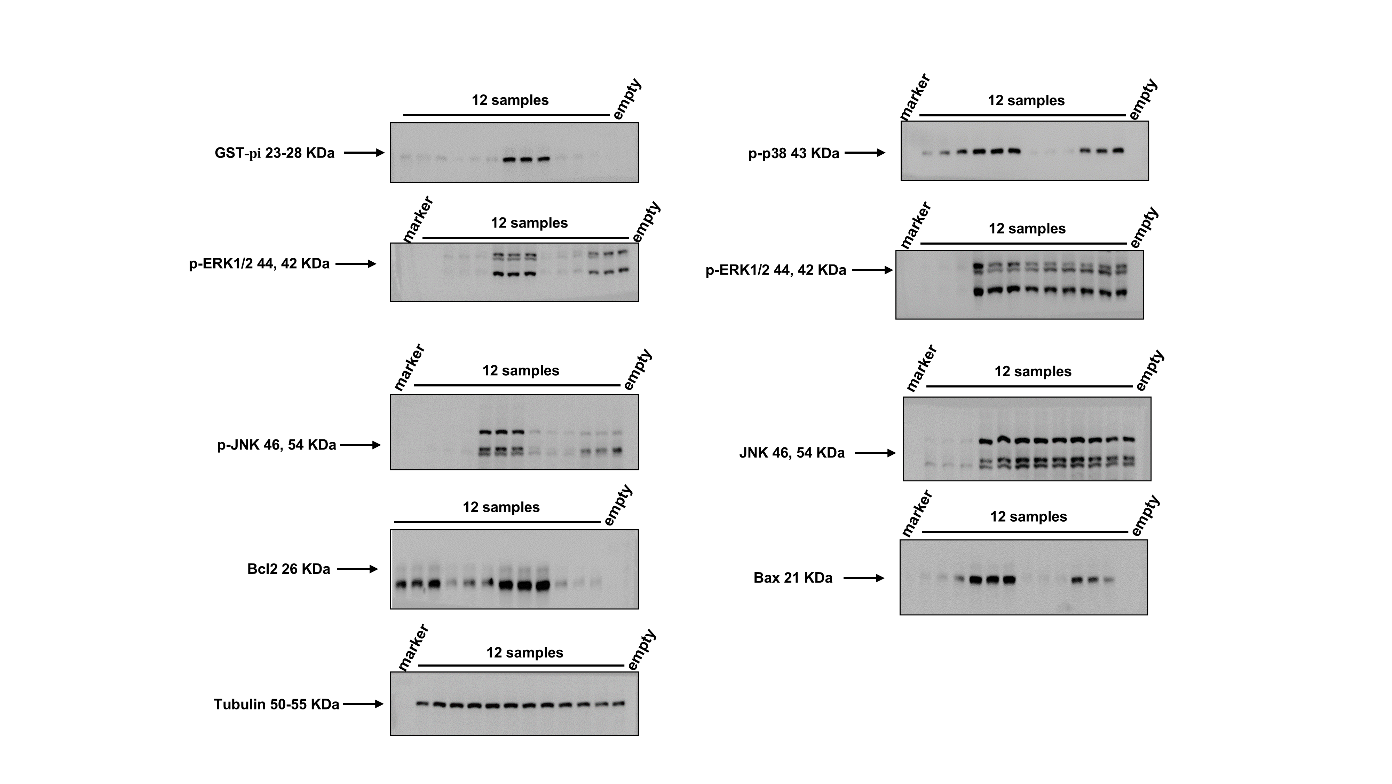

Supplement: Supplementary file 1 [file DataSheet2.docx]
