# Supplementary material for: Resveratrol and N-acetylcystein reduce hepatic steatosis but enhance initiation and progression of hepatocellular carcinoma by inhibiting GST-pi-MAPK axis in mice
Source: Front Pharmacol. 2025 Apr 28;16:1574039. doi: 10.3389/fphar.2025.1574039 (PMC12066552; doi:10.3389/fphar.2025.1574039)
Supplement: Supplementary file 2 [file DataSheet1.docx]

Supplementary Material

# Supplementary Figures and Tables

## Supplementary Figures
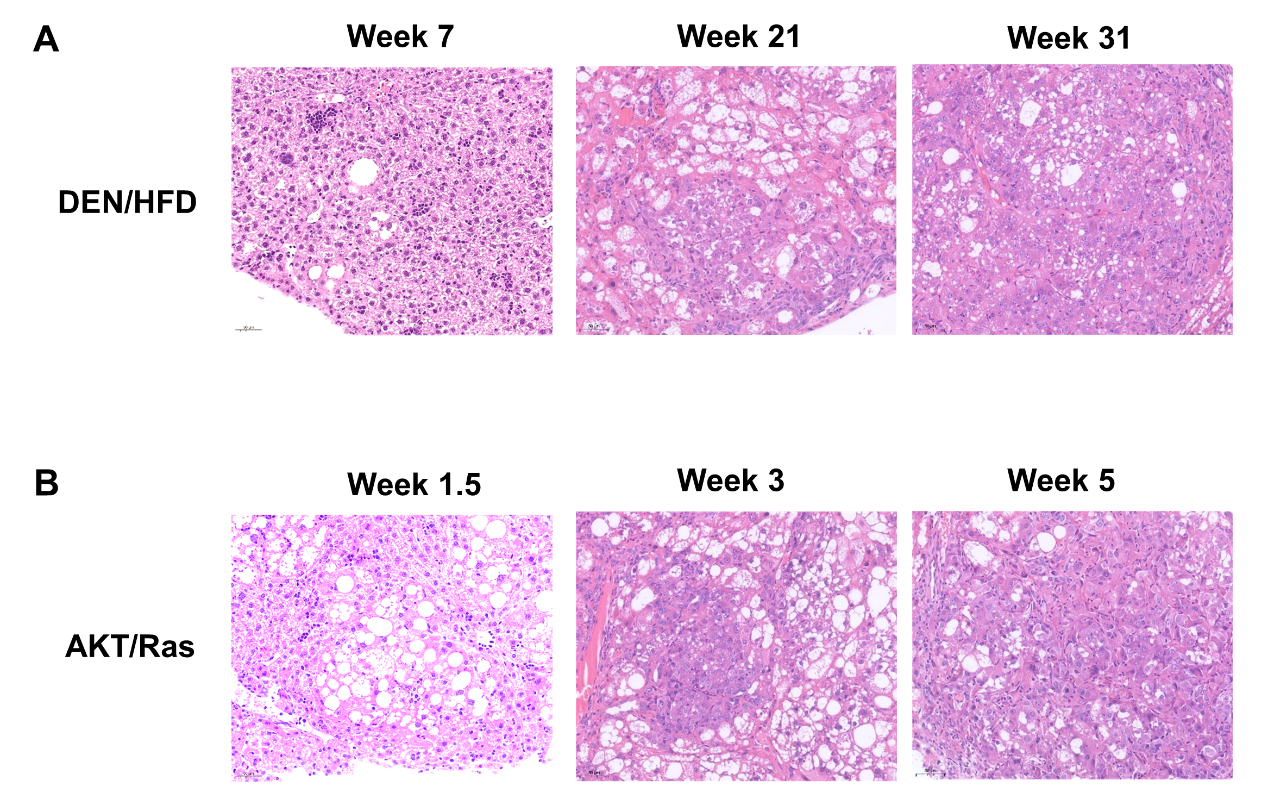


**Supplementary Figure 1.** Anatomy was performed on DEN/HFD (**A**) and AKT/Ras (**B**) model mice at three stages of tumor development, followed by H&E staining and microscopic photography of mouse liver tissue slices. Scale bars = 50 μm.


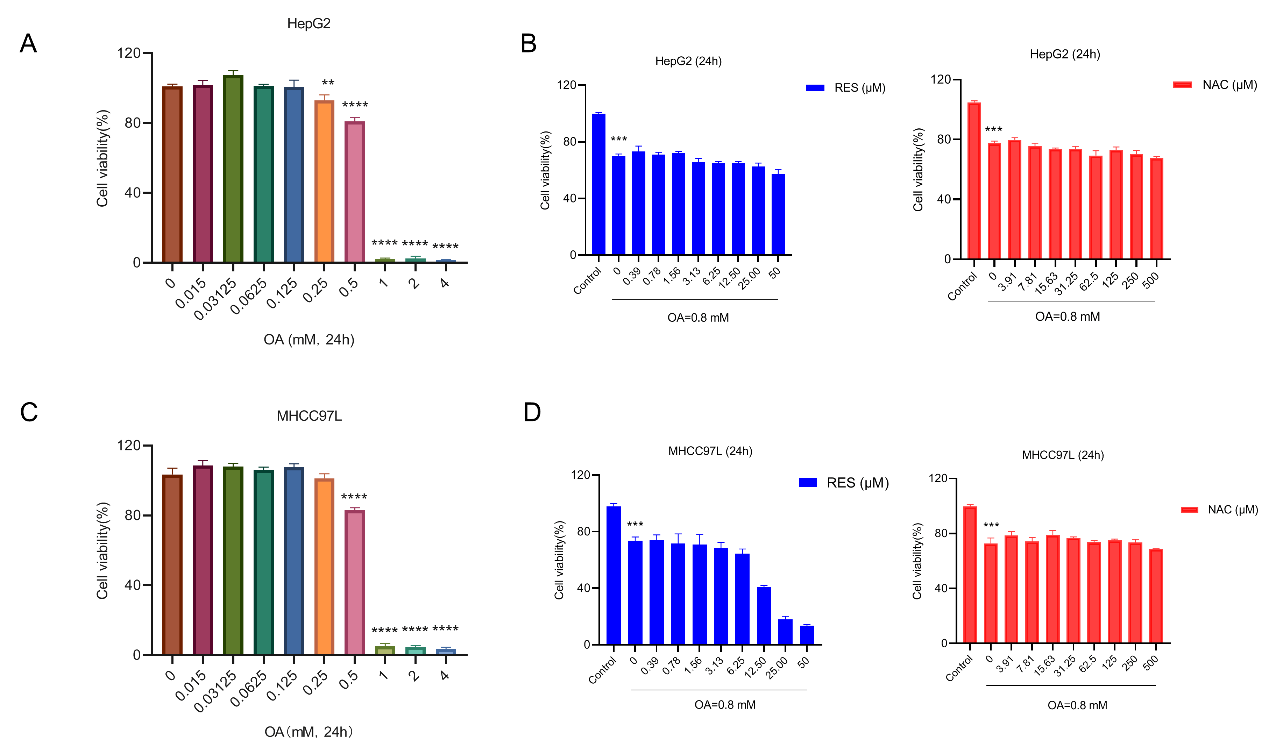


**Supplementary Figure 2.** **RES and NAC do not restore proliferation by OA treatment in a series of concentration.** (**A**) Cell viability of HepG2 cells. HepG2 cells treated with or without OA in a series of concentrations treatment for 24 h. (**B**) Cell viability of HepG2 cells. HepG2 cells treated with OA (0.8 mM) for 24 h with and without RES or NAC in a series of concentrations treatment. (**C**) Cell viability of MHCC97L cells. MHCC97L cells treated with or without OA in a series of concentrations treatment for 24 h. (**D**) Cell viability of of MHCC97L cells. MHCC97L cells treated with OA (0.8 mM) for 24 h with and without RES or NAC in a series of concentrations treatment.

## Supplementary Tables

**Table S1 Mice feed formula**

| **product** | **H10060** | |
| --- | --- | --- |
|  | Mass ratio g % | Energy ratio kcal % |
| protein | 26 | 20 |
| carbohydrate | 26 | 20 |
| fat | 35 | 60 |
| total |  | 100 |
| kcal/g | 5.24 |  |

| **product** | **H10060** | |
| --- | --- | --- |
| component | g | kcal |
| casein | 258.45 | 1033.80 |
| cystine | 3.88 | 15.52 |
| maltodextrin | 161.53 | 646.12 |
| sucrose | 88.91 | 355.64 |
| cellulose | 64.61 | 0 |
| Soybean oil | 32.31 | 290.79 |
| lard | 316.60 | 2849.40 |
| Mineral mixture M1002 | 12.92 | 0 |
| Calcium hydrogen phosphate | 16.80 | 0 |
| calcium carbonate | 7.11 | 0 |
| Potassium Citrate | 21.32 | 0 |
| Vitamin mixture V1001 | 12.92 | 51.68 |
| Choline Bitartrate | 2.58 | 0 |
| Edible blue dye | 0.065 | 0 |
| total | 1000 | 5242.95 |

| **product** | **H10010** | |
| --- | --- | --- |
|  | Mass ratio g % | Energy ratio kcal % |
| protein | 19.2 | 20 |
| carbohydrate | 67.3 | 70 |
| fat | 4.3 | 10 |
| total |  | 100 |
| kcal/g | 3.85 |  |

| **product** | **H10010** | |
| --- | --- | --- |
|  | g | kcal |
| casein | 189.58 | 758.32 |
| cystine | 2.84 | 11.36 |
| Corn Starch | 298.59 | 1194.36 |
| maltodextrin | 33.18 | 132.72 |
| sucrose | 331.77 | 1327.08 |
| cellulose | 47.40 | 0 |
| Soybean oil | 23.70 | 213.30 |
| lard | 18.96 | 170.64 |
| Mineral mixture M1002 | 9.48 | 0 |
| Calcium hydrogen phosphate | 12.32 | 0 |
| calcium carbonate | 5.21 | 0 |
| Potassium Citrate | 15.64 | 0 |
| Vitamin mixture V1001 | 9.48 | 37.92 |
| Choline Bitartrate | 1.90 | 0 |
| Edible blue dye | 0.047 | 0 |
| total | 1000 | 3845.70 |

**Table S2 Antibody Source information**

| **Antibody** | **Brand** | **Cat. No** |
| --- | --- | --- |
| Ki67 | Cell Signaling Technology | 9449S |
| Ki67 | proteintech | 27309-1-AP |
| γH2AX | Abcam | ab81299 |
| Histone H3 | Cell Signaling Technology | 9715T |
| GST-pi | proteintech | 15902-1-AP |
| Tubulin | proteintech | 66031-1-Ig |
| Phospho-p38 MAPK | Cell Signaling Technology | 4511-T |
| p-ERK1/2 | Cell Signaling Technology | 4370S |
| p-JNK | Cell Signaling Technology | 4668T |
| ERK1/2 | Cell Signaling Technology | 9102T |
| JNK | Cell Signaling Technology | 9252T |
| Bcl2 | proteintech | 26593-1-AP |
| Bax | proteintech | 50599-2-Ig |
